# An Internist’s Approach to a Case of Negative Pressure Pulmonary Edema: A Rare Cause of Noncardiogenic Pulmonary Edema

**DOI:** 10.7759/cureus.39587

**Published:** 2023-05-28

**Authors:** Renato Cerna-Viacava, Mohamed Ramzi Almajed, Julio Pinto Corrales

**Affiliations:** 1 Internal Medicine, Henry Ford Hospital, Detroit, USA; 2 Pulmonary and Critical Care Medicine, Henry Ford Hospital, Detroit, USA

**Keywords:** negative-pressure pulmonary edema, laryngospasm, acute hypoxic respiratory failure, postoperative hypoxia, post operative complication, invasive mechanical ventilation, non-cardiogenic pulmonary edema

## Abstract

Negative-pressure pulmonary edema (NPPE) is a rare cause of noncardiogenic pulmonary edema, which usually presents postoperatively. Its pathophysiology is mostly described as a profound negative intrathoracic pressure caused by an airway obstruction such as laryngospasm, which may occur during extubation. But, there are other hypotheses about it, such as catecholamines release causing an elevated hydrostatic pressure in the cardiopulmonary circuit and, consequently, a major capillary leak to the interstitium. Its natural course varies, from prompt recovery to intensive care unit escalation and prolonged mechanical ventilation. Although anesthesiologists often detect this condition, this case's objective is to bring awareness of this condition to internists as a potential differential diagnosis for hypoxia in the postoperative setting.

## Introduction

Negative pressure pulmonary edema (NPPE) is a rare cause of noncardiogenic pulmonary edema. Its pathophysiology is mostly described as a profound negative intrathoracic pressure caused by an airway obstruction such as laryngospasm, which may occur immediately after the extubation process [1]. But, there are other hypotheses about it, such as catecholamines release causing an elevated hydrostatic pressure in the cardiopulmonary circuit and, consequently, a major capillary leak to the interstitium. Also, there are hypotheses of extrinsic compression of the endotracheal tube caused by strong laryngeal reflexes and reflex tube biting from the patients. Its natural course is variable, from prompt recovery to intensive care unit escalation and prolonged mechanical ventilation.NPPE varies in severity of presentation, and there are no specific indicators of decompensation or prognosis. A significant portion of patients with NPPE recovers with observation and oxygen supplementation. However, some patients require advanced interventions, including support with mechanical ventilation [2].

NPPE is frequently identified and encountered by anesthesiologists after surgery. However, some cases of NPPE have a delayed presentation and manifest at a point when anesthesiologists are no longer directly involved in patient care. Therefore, other physicians must consider NPPE among the differential diagnoses for postoperative patients who develop shortness of breath and hypoxia.

We present the case of a healthy patient who was intubated with general anesthesia for elective orthopedic surgery and developed NPPE in the postoperative period. He was admitted to the hospital's general practice unit, where he recovered with oxygen supplementation and medical management. This is an exemplary case that serves as a reminder of the complications such as NPPE, which can develop in an otherwise healthy patient who has been intubated. Physician awareness of complications that may arise after intubation is critical for preventing serious outcomes and is particularly important in the era of COVID-19, where the need for intubation is increasingly common.

## Case presentation

A 68-year-old man with a height of 1.88 m (6'2" ft), 88 kg (202 lbs), a body mass index of 24.7 kg/m^2^, and an unremarkable medical history underwent an elective orthopedic surgery during which he had repair of a rotator cuff tear. His social history was significant for occasional alcohol use; he did not smoke or use illicit substances. The operation was performed with the patient under general anesthesia and endotracheal intubation, which was smooth with no unexpected complications; lidocaine, fentanyl, rocuronium, and propofol were administered. The preoperative and intraoperative courses were uneventful, and the operation was successful. Before endotracheal intubation, the patient's respiratory status was completely normal; not in any respiratory distress or using any oxygenation delivery system. Also, immediately after extubation, the patient was placed on minimal oxygen supplementation via nasal cannula but was not having signs of respiratory distress. During the extubation process, the anesthesiologist did not provide evidence of any signs of airway obstruction.

**Figure 1:**
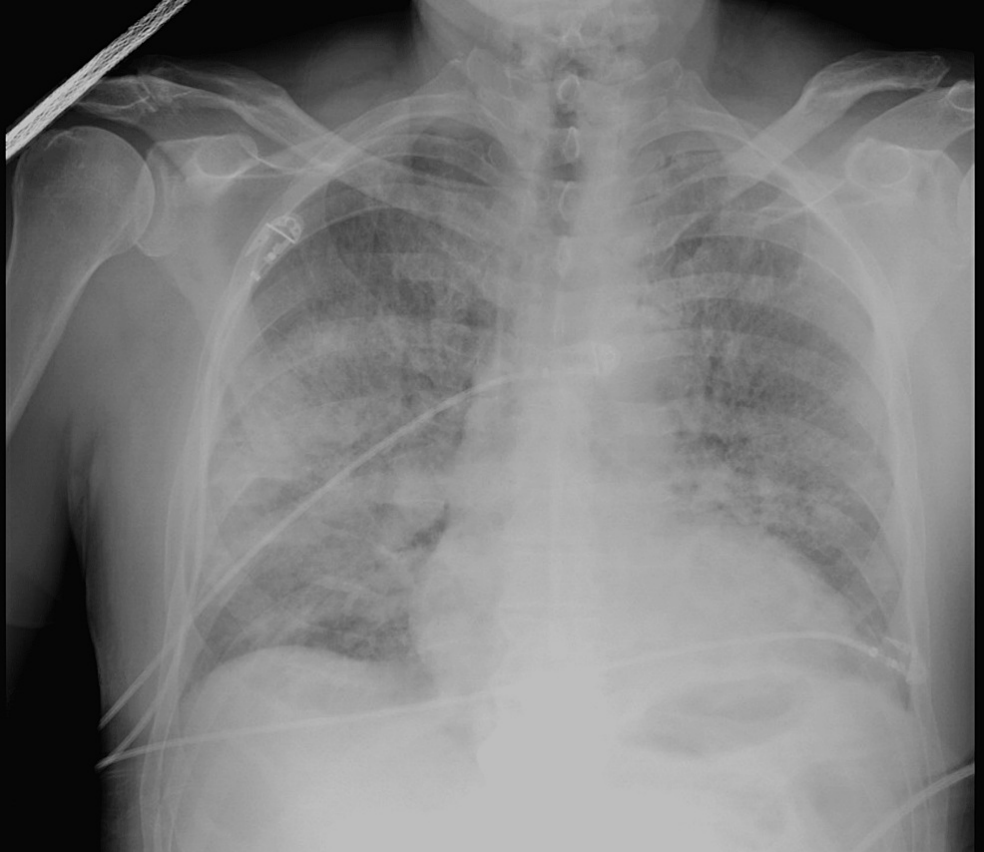
Initial Chest X-Ray.

Within one hour after surgery, the patient developed sudden hypoxia with an oxygen saturation of 85% on room air. He was initially unresponsive, and auscultation by the anesthesiologist was remarkable for adequate air entry with harsh breath sounds bilaterally. Oxygen supplementation was administered via a nasal cannula; the patient received naloxone, albuterol, and furosemide, after which he became alert but had persistent hypoxia. A chest x-ray was obtained, which showed patchy confluent airspace disease most prominent in the right midlung (Figure 1).

**Figure 2:**
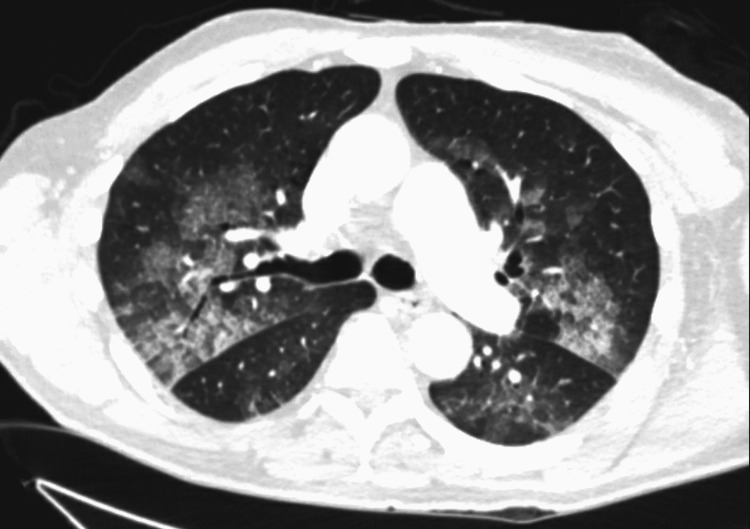
Ground glass opacities in Chest CT

A computed tomography (CT) scan of the chest showed scattered ground-glass opacities throughout the lung lobes with areas of "crazy-paving"; no findings were suggestive of pleural effusion or pneumothorax (Figure 2).

The patient reported persistent shortness of breath and remained hypoxic. He was subsequently transferred to the pulmonology medical service for further treatment.

**Figure 3:**
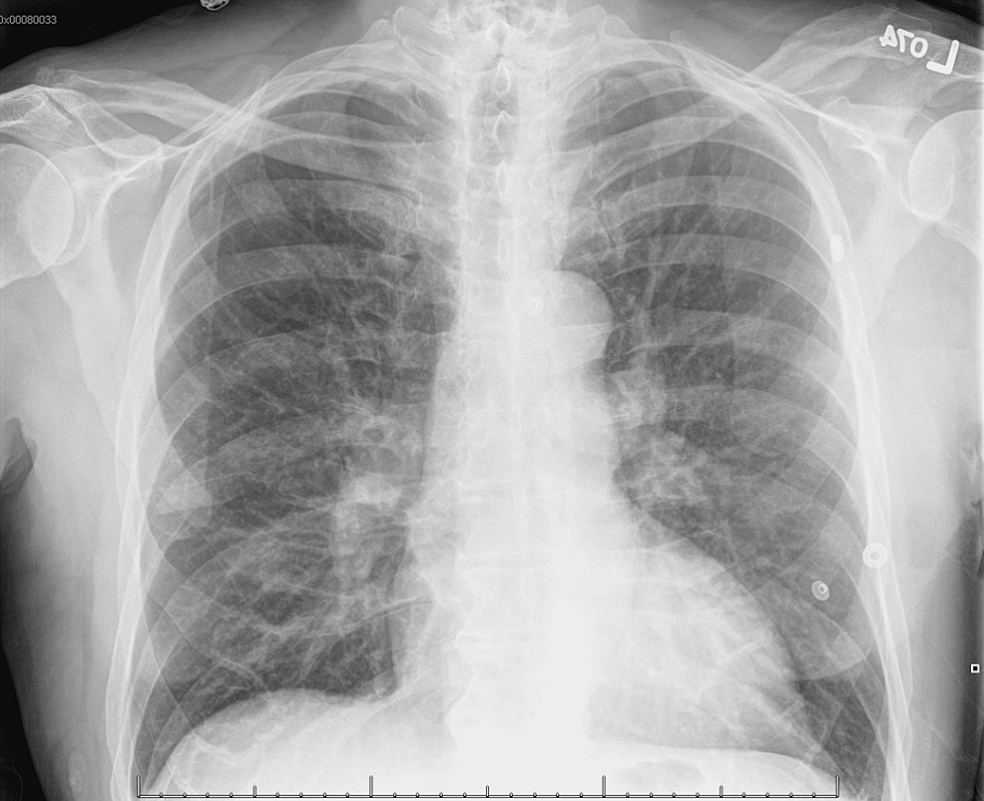
Final Chest X-Ray.

The patient remained on supplemental oxygen via nasal cannula at 2L/min over 24 hours; oxygen saturation remained above 96% over this period. His vital signs were unremarkable, and he reported improvement in his symptoms. On the second day of hospitalization, a repeat chest X-ray was obtained, which showed significant improvement in the bilateral airspace opacities with mild persistence prominence of the interstitium, suggesting resolving pulmonary edema (Figure 3).

Ambulatory pulse oximetry was then carried out, and the patient could ambulate on room air without signs of respiratory distress; his oxygen saturation remained above 92% without oxygen supplementation.

Further workup was done to determine the etiology of the patient's hypoxia. His electrocardiogram showed sinus tachycardia without a waveform or segment abnormalities, and his serum troponin and b-type natriuretic peptide were within normal limits. Respiratory specimens were positive for respiratory syncytial virus and negative for influenza A, influenza B, and SARS-CoV-2. Of note, the patient did not have upper respiratory tract infection symptoms. Autoimmune investigations were negative for antinuclear antibodies, antineutrophil cytoplasmic antibodies, rheumatoid factor, anti-CCP antibodies, anti-Ro and anti-La antibodies, and anti-centromere and anti-Scl-70 antibodies.

In the context of our patient's endotracheal intubation and in the complete absence of cardiovascular, pulmonary, and autoimmune etiologies explaining his presentation, a diagnosis of negative pressure pulmonary edema was made.

## Discussion

NPPE is a rare cause of non-cardiogenic pulmonary edema due to acute upper airway obstruction. It typically occurs in the setting of endotracheal intubation or the use of laryngeal masks, aerodigestive tract tumors, thyroid gland tumors, infections such as epiglottitis, trauma, and any conditions which can develop stridor due to airway obstruction [3]. In the hospital, NPPE usually occurs after extubation in the postoperative period and is commonly identified by anesthesiologists while patients are emerging from anesthesia. Symptom onset, however, may be delayed and has been described as occurring as late as 2.5 hours post-extubation [4-5]. The description of delayed presentation of NPPE necessitates greater awareness among physicians to facilitate early identification and prompt initiation of treatment.

The pathophysiology underlying the development of NPPE involves an inciting event that triggers significant negative intrathoracic pressure. In post-extubation NPPE, the presence of a foreign object in the upper airway can trigger laryngospasm in which a forced inspiratory effort is made against a closed glottis, thus causing negative intrathoracic pressure that potentiates blood flow to the right heart and delivery of blood to the pulmonary circulation [6]. This acute increase in pulmonary flow triggers dilation of the vasculature that lowers interstitial hydrostatic pressure, causing an efflux of intravascular fluid into the interstitium at a rate greater than the lymphatic drainage [7]. The subsequent ventilation-perfusion (V/Q) mismatch and poor gas exchange cause hypoxemia and triggers compensatory systemic hypertension that increases afterload and further promotes filtration; these maladaptive hemodynamics propagate interstitial and alveolar edema. Refractory cases have been reported to lead to diffuse alveolar hemorrhage and death [8].

Patients with NPPE present with an abrupt sensation of dyspnea with marked hypoxia, as was seen in our patient. Some patients develop hemoptysis and orthopnea [9]. Chest x-ray findings consistent with the diagnosis include bilateral centralized pulmonary infiltrates, wide vascular pedicle, and the normal cardiothoracic ratio [10]. A "crazy-paving" pattern on a CT scan of the chest, as seen in our patient, is a nonspecific finding often seen in pulmonary edema and interstitial fibrosis [11]. In the typical clinical context, anesthesiologists often accurately identify NPPE postoperatively and institute care. However, if the temporal association between extubation and symptom development is not recognized, this diagnosis may be overlooked or remain unclear.

Treatment of NPPE involves relieving the upper airway obstruction to reverse the pathophysiologic hemodynamics, alleviate the pulmonary edema, and correct the hypoxemia, such as positive airway pressure delivery systems (BiPAP, CPAP) or high-flow nasal cannula to prevent reintubation. Patients whose inciting cause of the obstruction is laryngospasm from extubation have a reversible obstruction that generally self-resolves, and these patients merely require support with supplemental oxygen. These patients are commonly noted to have a perioperative course significant for biting the endotracheal tube [2]. Patients with significant laryngospasm or an anatomical obstruction need further support with noninvasive positive pressure ventilation, and those who do not respond favorably require bypassing of the upper airway by endotracheal intubation or tracheostomy and mechanical ventilation [12]. Low tidal volumes are preferred in these patients, as lung-protective ventilation prevents the development of ventilator-associated lung injury [13].

Additional supportive measures may hasten the resolution of pulmonary edema and improve outcomes. Muscle relaxants such as rocuronium are used to alleviate laryngospasm [14]. B2-agonist bronchodilators have been consistently used, as they are thought to improve alveolar fluid clearance [15]. Diuretics, used only in the absence of hypovolemia, reduce the rate of fluid efflux into the pulmonary interstitium and accelerate the radiographic resolution of edema [16]. Early identification of ongoing respiratory distress, prompt administration of supportive care, and escalation of care when necessary are the pillars of management for NPPE.

## Conclusions

NPPE is a condition that develops in patients undergoing surgical procedures involving endotracheal intubation or laryngeal masks as a possible complication explained by negative intrathoracic pressures causing capillary leaks and, consequently, pulmonary edema. Causes of this pathophysiologic mechanism are acute airway obstruction such as laryngospasm after extubation, tube biting, or catecholamine release. This case report aims to bring awareness among non-anesthesiologists, such as Internal Medicine, to have NPPE as part of the differential diagnosis of patients who develop hypoxia rapidly after a surgical procedure so this condition can be identified and treated promptly. Continuous monitoring of respiratory status with supportive treatment and oxygen supplementation is key to rapidly improving symptoms and resolving radiographic abnormalities, especially in an otherwise healthy patient presenting with NPPE.
